# Supplementary material for: Effect of Influenza Vaccination on Rate of Influenza Virus Infection in Chinese Military Personnel, 2015–2016: A Cluster Randomized Trial
Source: Vaccines (Basel). 2023 Aug 31;11(9):1439. doi: 10.3390/vaccines11091439 (PMC10534408; doi:10.3390/vaccines11091439)
Supplement: Supplementary file 1 [file vaccines-11-01439-s001.zip › vaccines-2542839-supplementary.pdf]

vaccines

MDPI

Table S1. Definition of severe influenza.

No.	Criteria of severe influenza *
1	Altered mental status, defined as obtunded, lethargy, restlessness or convulsions;
2	Dyspnea and/or increased respiratory rate (>30 breaths per minute);
3	Severe vomiting, diarrhea, or dehydration;
4	Oliguria (<400ml per 24 hours) or acute renal failure;
5	Arterial blood pressure <90/60 mmHg;
6	Arterial partial pressure of oxygen (PaO2) <60 mmHg or PaO2/FiO2 <300;
7	Chest X-ray showing bilateral or multi-lobar infiltrates, or pulmonary infiltrates expanding ≥50% within 48 hours of admission;
8	Abnormal and high levels of creatine kinase and creatine kinase isoenzyme;
9	Or organ insufficiency or failure, or acute exacerbation of underlying diseases.

Note: * Criteria were adopted from the guidelines issued by a former Ministry of Health, 2011. Severe influenza was a person with diagnosed influenza and had at least one severe symptom or syndrome of above in the table.

Table S2. Clinical characteristics of participants with laboratory-confirmed influenza.

Signs/symptoms	Vaccine group (n=18)	Control group (n=87)	p-value
Body temperature, °C, median (IQR)	38.5 (38.4-38.9)	38.6 (38.1-39.1)	0.697
Respiratory rate, breaths/min, median (IQR)	19 (17-20)	20 (18-20)	0.538
Fever, no. (%)	18 (100)	87 (100)	1
Cough, no. (%)	11 (61.1)	52 (59.8)	1
Sore throat, no. (%)	10 (55.6)	32 (36.8)	0.187
Productive cough, no. (%)	9 (50.0)	46 (52.9)	1
Fatigue, no. (%)	5 (27.8)	43 (49.4)	0.121
Runny nose, no. (%)	2 (11.1)	29 (33.3)	0.087
Headache, no. (%)	1 (5.6)	33 (37.9)	0.006
Myalgia, no. (%)	0 (0)	35 (40.2)	0.001
Abdominal pain, no. (%)	4 (22.2)	16 (18.4)	0.744
Diarrhea, no. (%)	2 (11.1)	20 (23)	0.351
Dyspnea, no. (%)	0 (0)	0 (0)	1
Antibiotic treatment, no. (%)	1 (5.6)	2 (2.3)	0.435

Abbreviations, IQR=interquartile range.

Table S3. Hemagglutination Inhibition Antibody Responses Before and After influenza Vaccination, by four influenza virus strains (A/H3N2, A/H1N1, B Yamagata lineage, and B Victoria lineage).

Influenza virus strains	Indicators	Hemagglutination Inhibition Antibody (at Day 0)			Hemagglutination Inhibition Antibody (at Day 21)		
		Vaccine group (n=67)	Control group (n=82)	P-valu	Vaccine group (n=67)	Control group (n=82)	P-value
A/H1N1							
	Geometric mean titers, (95% CI)	30.9 (23.9-39.9)	20.7 (16.3-26.3)	0.026	237.1 (183.7-306.0)	21.6 (16.7-28.0)	<0.001
	Seroconversion rate. (%, 95% CI)	-	-	-	68.7 (56.0-79.1)	0.0 (0.0-5.6)	<0.001
	Sero-protection rate (%, 95% CI)	55.2 (42.6-67.2)	41.5 (30.9-52.9)	0.094	98.5 (90.0-99.9)	43.9 (33.1-55.3)	<0.001

	Geometric mean increase, (95% CI)	-	-	-	7.7 (4.6-12.8)	1.0 (0.6-1.7)	<0.001
A/H3N2							
	Geometric mean titers, (95% CI)	45.8 (37.9-55.3)	61.0 (51.4-72.5)	0.026	385.5 (303.2-490.1)	64.8 (54.3-77.3)	<0.001
	Seroconversion rate. (%, 95% CI)	-	-	-	85.1 (73.8-92.2)	0.0 (0.0-5.6)	<0.001
	Sero-protection rate (%, 95% CI)	77.6 (65.5-86.5)	86.6 (76.8-92.8)	0.151	100.0 (93.2-100.0)	84.1 (74.0-91.0)	0.002
	Geometric mean increase, (95% CI)	-	-	-	8.4 (5.5-12.9)	1.1 (0.7-1.5)	<0.001
B/Yamagata							
	Geometric mean titers, (95% CI)	22.0 (17.1-28.2)	19.5 (15.1-25.2)	0.517	249.6 (196.0-318.0)	24.1 (18.8-30.8)	<0.001
	Seroconversion rate. (%, 95% CI)	-	-	-	86.6 (75.5-93.3)	9.8 (4.6-18.8)	<0.001
	Sero-protection rate (%, 95% CI)	43.3 (31.4-55.9)	36.6 (26.4-48.0)	0.406	97.0 (88.7-99.5)	47.6 (36.5-58.8)	<0.001
	Geometric mean increase, (95% CI)	-	-	-	11.4 (7.0-18.6)	1.2 (0.7-2.0)	<0.001
B/Victoria							
	Geometric mean titers, (95% CI)	4.6 (4.0-5.3)	5.3 (4.5-6.3)	0.204	9.7 (7.9-11.9)	4.9 (4.1-5.8)	<0.001
	Seroconversion rate. (%, 95% CI)	-	-	-	9 (3.7-19.1)	1.2 (0.1-7.5)	0.091
	Sero-protection rate (%, 95% CI)	0.0 (0.0-6.8)	2.4 (0.4-9.4)	0.568	11.9 (5.7-22.7)	4.9 (1.6-12.7)	0.203
	Geometric mean increase, (95% CI)	-	-	-	2.1 (1.5-3.0)	0.9 (0.7-1.3)	<0.001
